# Sensor-based characterization of daily walking: a new paradigm in pre-frailty/frailty assessment

**DOI:** 10.1186/s12877-020-01572-1

**Published:** 2020-05-06

**Authors:** Danya Pradeep Kumar, Nima Toosizadeh, Jane Mohler, Hossein Ehsani, Cassidy Mannier, Kaveh Laksari

**Affiliations:** 1 Department of Biomedical Engineering, University of Arizona, Tucson, AZ USA; 2 Arizona Center on Aging, Department of Medicine, University of Arizona, Tucson, AZ USA; 3 Department of Aerospace and Mechanical Engineering, University of Arizona, Tucson, AZ USA

**Keywords:** Frailty, Daily physical activity, Wearable sensors, Continuous walking, Performance parameters

## Abstract

**Background:**

Frailty is a highly recognized geriatric syndrome resulting in decline in reserve across multiple physiological systems. Impaired physical function is one of the major indicators of frailty. The goal of this study was to evaluate an algorithm that discriminates between frailty groups (non-frail and pre-frail/frail) based on gait performance parameters derived from unsupervised daily physical activity (DPA).

**Methods:**

DPA was acquired for 48 h from older adults (≥65 years) using a tri-axial accelerometer motion-sensor. Continuous bouts of walking for 20s, 30s, 40s, 50s and 60s without pauses were identified from acceleration data. These were then used to extract qualitative measures (gait variability, gait asymmetry, and gait irregularity) and quantitative measures (total continuous walking duration and maximum number of continuous steps) to characterize gait performance. Association between frailty and gait performance parameters was assessed using multinomial logistic models with frailty as the dependent variable, and gait performance parameters along with demographic parameters as independent variables.

**Results:**

One hundred twenty-six older adults (44 non-frail, 60 pre-frail, and 22 frail, based on the Fried index) were recruited. Step- and stride-times, frequency domain gait variability, and continuous walking quantitative measures were significantly different between non-frail and pre-frail/frail groups (*p* < 0.05). Among the five different durations (20s, 30s, 40s, 50s and 60s), gait performance parameters extracted from 60s continuous walks provided the best frailty assessment results. Using the 60s gait performance parameters in the logistic model, pre-frail/frail group (vs. non-frail) was identified with 76.8% sensitivity and 80% specificity.

**Discussion:**

Everyday walking characteristics were found to be associated with frailty. Along with quantitative measures of physical activity, qualitative measures are critical elements representing the early stages of frailty. In-home gait assessment offers an opportunity to screen for and monitor frailty.

**Trial registration:**

The clinical trial was retrospectively registered on June 18th, 2013 with ClinicalTrials.gov, identifier NCT01880229.

## Background

Among the population who are 60 years or older, frailty is a highly recognized syndrome that is associated with decline in function and reserve across multiple physiologic systems [1–5]. Frailty is characterized by a high vulnerability to adverse health outcomes such as disability, falls, hospitalization, institutionalization, and mortality [1]. Reduction or impairment of physical function is a prime indicator of frailty [2], and frailty is one of the major reasons for falls in old age [6–12]. Many definitions of frailty have been proposed: Fried et al. used five criteria (slowness, exhaustion, weakness, low-activity and weight-loss) to identify frailty [2]; Rockwood et al. developed a frailty index based on impairments in cognitive status, mood, motivation, communication, mobility, balance, bowel and bladder function, activities of daily living, nutrition, social resources and number of comorbidities [13]; Mitnitski et al. constructed a frailty index based on 20 deficits as observed in a structural clinical examination based on the comprehensive geriatric assessment (CGA) [14]; Jones et al. also based their frailty index based on CGA which included 10 standard domains to construct a three level frailty index permitting risk stratification of future adverse outcomes [15]; and Chin et al. compared three different working definitions of frailty – inactivity plus low energy intake, inactivity plus weight-loss and inactivity plus low body mass index [16]. Although many definitions of frailty have been proposed, we use Fried’s frailty criteria as the most commonly implemented frailty assessment tool in our study. However, there is currently no objective method for assessing frailty that incorporates assessment of daily physical activity (DPA).

DPA data has been recently used to assess physical function, especially with the help of wearable sensor technology. Using wearable devices, it is possible to continuously measure DPA in the least intrusive manner and for longer durations of time in the participants’ natural environment. Among several DPA, motion analysis of the trunk during walking is known to provide insights regarding neuromuscular deficits associated with frailty and aging [12]. In our previous studies, among DPA measures (walking, standing, sitting, and lying), quantitative parameters related to walking, such as total walking duration and maximum number of steps, best discriminated between non-frail and pre-frail groups, with highest effect sizes for the number of steps and the percentage of walking duration within a 24-h time period [17]. While promising, we found that none of these outcomes (or their combination in a multinomial logistic analysis) could significantly discriminate between frailty groups when adjusted for age [17].

In this study, we aimed to improve detection of frailty-related neuromuscular deficits based on gait performance parameters derived from unsupervised DPA. Previous studies have used trunk motion data from supervised in-lab gait tests to characterize sensorimotor gait performance among frail elders including gait variability, asymmetry, initiation, and irregularity [11, 12, 18–22]. We hypothesized that using more robust measures of unsupervised DPA gait performance such as gait variability, asymmetry and irregularity (instead of number of steps) it would be possible to distinguish between non-frail and pre-frail/frail older adults.

## Methods

This observational cross-sectional study was performed at Arizona Center on Aging, Tucson, AZ. Participants in this study were from primary, secondary, and tertiary health-care settings within our academic network and also from community providers and aging service organizations. DPA was recorded from eligible volunteers for 48 h and the walking data from the DPA was processed to study the gait performance parameters and associate these characteristics with frailty.

### Participants

Older adults (65 years or older), without severe mobility disorder and the ability to walk at least 10 m with or without an assistive device, were considered eligible for the study. Participants with cognitive-impairment (screened by a Mini-Mental State Examination (MMSE) [23] score of < 23) or terminal illness were excluded. All the eligible participants signed a written consent form according to the principles expressed in the Declaration of Helsinki [24], approved by the Institutional Review Board of the University of Arizona.

### Demographic and clinical measures

The recorded clinical measures included self-reported history of falls, use of assistive device and the number of prescriptions. Interviewer-administered questionnaires included the MMSE, Mobility-Tiredness Scale [25], Center for Epidemiologic Studies Depression Scale (CES-D) [26], Falls Efficacy Scale-International (FES-I) [27], and Barthel Activity of Daily Living (ADL) Scale [28].

### Frailty assessment

Frailty was assessed using the five criteria proposed by Fried et al. [2], including: self-reported weight loss, weakness measure by the grip strength, self-reported exhaustion, slowness measure by the walking test, and self-reported low energy expenditure. A score of one point was given for each criterion recorded, totaling a score in the range of 0–5. Frailty was categorized as follows: non-frail (score 0), pre-frail (score 1–2), and frail (score 3–5).

### Sensor-based daily physical activity assessment

DPA was quantified for two consecutive days (48 h) using a tri-axial accelerometer sensor (PAMSys, BioSensics Cambridge, MA, USA) fixed in a tee-shirt, with a device pocket located at the sternum. PAMSys is a small (5.1 × 3 × 1.6 cm), light (24 g) recording system containing inertial sensors. PAMSys is an ambulatory system designed and developed for human motion analysis using a kinematic sensor. It uses one kinematic sensor placed on the chest and is capable of accurately identifying and recording postural transitions (sit-to-stand and stand-to-sit), laying, walking, and standing [29–31]. PAMSys uses discrete wavelet transformation techniques based on a previously validated algorithm (PAMWare, BioSensics Cambridge, MA, USA) to identify walking bouts [32–34]. Using this method, walking bouts were defined by a minimum of three successive steps [29], where steps were estimated by the detection of an acceleration peak beyond a predefined threshold after filtering the signal [31]. Using this software several gait parameters were derived including: the duration of walking, walking bout times (duration of each walking episode), number of steps per walking bout, and walking cadence per bout. PAMWare is 87% sensitive and 87% specific for gait detection [29, 30]. In the current study, we used raw vertical acceleration data recorded by the PAMSys tri-axial accelerometer sensor, as well as the timing of walking bouts from the PAMWare software to extract gait performance parameters within continuous walking bouts.

### Continuous walking bouts

Previous studies that explored the gait characteristics of non-disabled adults for 2 weeks to define walking duration, found that 81% of all walking bouts lasted about 60 s [35, 36]. Further, previous studies suggested that a duration of 60-s continuous data would provide a reliable sample for nonlinear dynamic analysis [37]. Accordingly, in our study, we used continuous walking bouts of 60 s or longer for the extraction of gait performance parameters. In addition to the 60s analysis, all the gait performance parameters were extracted for 20s, 30s, 40s and 50s lengths of continuous walking bouts to investigate the effect of continuous walk length on data analysis and results. Gait performance parameters including time- and frequency-domain gait variability, gait asymmetry, and gait irregularity were extracted from these continuous walking bouts of all the durations, with no pauses longer than 1.7 s between gait cycles [17]. Allowable 1.7 s pause between gait cycles was conservatively selected based on the average plus standard deviation stride time duration observed in frail participants [17]. All the sensor-based gait performance outcome measures are shown in Table 1.

**Table 1:** Sensor-based outcome measures

Parameter	Description	Reference
Step/stride time	Time-interval between two consecutive/alternate acceleration peaks	
*Gait Variability*		
Step/stride time variability	Coefficient of variation (%), standard deviation of step/stride time over mean step/stride time	[17]
PSD max	Maximum height of the PSD distribution curve representing the amount of walking that occurs at the dominant frequency	[38, 39]
PSD width	The width of the PSD curve at half of the maximum height representing the range of walking frequencies	[38, 39]
PSD slope	The slope of the PSD curve from the peak to the width representing the variability of walking.	[38, 39]
Dominant frequency	The frequency at which the PSD curve attains its peak, representing the frequency at which most of the walking cycles occur	[38, 39]
*Gait Asymmetry*		
	Unbiased auto-correlation coefficients of gait signal, representing left-right step coordination	[11, 40]
*Gait Irregularity*		
	Sample entropy, representing the predictability of walking cycles	[41–43]
*Continuous Walking Quantitative Measures*		
No. of continuous walks	Total number of continuous walks in the 48 h duration	
Total continuous walking duration	Total duration of continuous walks in the 48 h duration	
Max walking bout	Maximum duration of continuous walking in 48 h	
Max no. of continuous steps	Maximum number of continuous steps in the longest duration continuous walking bout in 48 h	
Walking bout variability	Coefficient of variation (%), standard deviation of walking bouts over mean walking bout	
Duration of non-continuous walks (% of total walking duration)	Duration of walks which were not continuous for 60s or longer (total duration of 60s walking minus continuous walking with no pause)	

*PSD* Power Spectral Density

For each continuous walking bout, the raw vertical acceleration signal extracted from the PAMSys sensor was filtered using a second order Butterworth filter (cut-off frequency of 2.5 Hz [44]), and the peaks of the filtered acceleration signal were detected using a peak-detection algorithm. The time-interval between two consecutive peaks was defined as the step-time, and the time-interval between alternate peaks was defined as the stride-time.

#### Gait variability

We defined gait variability as the stride-to-stride fluctuation in gait cycles, which has been associated with high risk of fall and cognitive impairments in elders [6, 45–47]. Gait variability reflects inconsistency in physiological systems that regulate walking, including neuromuscular, reflexive postural control, and cardiovascular systems [48]. We used two methods to assess gait variability: 1) step- and stride-time variability using time-domain; and 2) power spectral density (PSD) using frequency-domain analysis [49, 50]. Step- or stride-time variability was calculated as the coefficient of variation of the series of step- or stride-times for each continuous walking bout. For PSD analysis, the power spectrum of the acceleration data was calculated using Welch’s averaged modified periodogram method [38], to represent the frequency components of the acceleration signal [51]. We used a window size of 512 samples and an FFT length of 2-times the next higher power of the window size [38]. An overlap of 50% was considered between the windows. The locomotion band between 0.5–3.0 Hz was analyzed [38]. PSD components were extracted from the raw acceleration signal, including maximum PSD peak, PSD width (full width at half maximum height), PSD slope (PSD width to the peak) and dominant walking frequency. A higher variability in walking was identified by a shorter and wider PSD peak.

#### Gait Asymmetry

When gait becomes less automatic due to sarcopenia and cognitive aging, left-right step coordination may require more effort, especially among frail individuals [17, 37, 52]. Further, studies showed that no strong association between gait variability and asymmetry exists, suggesting that asymmetry reflects an independent measure of gait impairments due to distinct pathological causes [17, 52]. Here, step asymmetry was obtained from the autocorrelation function of the vertical acceleration signal [17, 52], represented by a sequence of autocorrelation coefficients over increasing time lags.

1 $$ Asymmetr{y}_1=\frac{A{d}_1}{A{d}_2},\kern0.5em Asymmetr{y}_2=\frac{\mid A{d}_1-A{d}_2\mid }{\max \left(A{d}_1,A{d}_2\right)} $$

where *Ad*_1_ and *Ad*_2_ are the prominence of the first and the second peaks respectively after the central (zero lag) peak [53].

#### Gait irregularity

Results from supervised gait studies showed that irregularity measures can describe predictability of walking cycles, which can be influenced by both neurological and neuromuscular diseases [18, 54–56]. Further, within in-lab settings, it has been demonstrated that gait irregularity can differ between non-frail and pre-frail older adults [21]. We used Sample Entropy (SampEn) assessment defined as Eq. (2), where *A* was defined as the number of matches in the filtered acceleration signal length *m* + 1 (distance function smaller than tolerance : *d*[*X*_*m* + 1_(*i*), *X*_*m* + 1_(*j*)] < *r*) and *B* as the number of matches of length *m*: (*d*[*X*_*m*_(*i*), *X*_*m*_(*j*)] < *r*) [41, 57–60].

2 $$ SampEn=-\log \left(\frac{\left({\sum}_{i=1}^{N-m}{A}_i\right)}{\left({\sum}_{i=1}^{N-m}{B}_i\right)}\right)=- logA/\mathrm{B} $$

The time-delay of the signal was calculated using mutual information method for all the continuous walks [61], and the average time-delay of all the continuous walks was used to calculate the SamplEn for each volunteer. We used embedding dimension m = 3, and tolerance *r* = 0.2 times the standard deviation of the signal, which are commonly used to compute sample entropy of gait signal [41, 57–60].

#### Continuous walking quantitative measures

In addition to the above-mentioned features, we extracted the following parameters in each continuous walking event: maximum walking bout, maximum number of continuous steps, walking-bout variability (coefficient of variation in walking bouts duration within 48 h), and total duration of continuous walks. Of note, the parameters extracted here were only obtained for continuous walking events with no pause of 1.7 s or longer, as described above. Duration of non-continuous walk (total duration of 60s walking minus 60s continuous walking with no pause) was obtained as a percentage of total duration of walking from the PAMsys sensor.

### Statistical analysis

Separate analysis of variance (ANOVA) models were performed to compare sociodemographic parameters between the three Fried frailty groups. To explore differences in gait performance parameters among frailty categories, univariate ANOVA models were used with each gait performance parameter as the dependent variable, and the Fried frailty categories (non-frail and pre-frail/ frail) as the independent variable. Subsequently, gait performance parameters were used in a single multivariable nominal logistic model to assess the association between frailty categories and DPA gait performance parameters. In this model we combined pre-frail and frail groups, due to the limited number of frail participants. The model was developed following these steps: 1) univariate nominal logistic model analysis of the gait performance parameters as independent variables was performed. Gait performance parameters with significant association with frailty were considered for subsequent steps; 2) collinearity between the various gait performance parameters was tested using the variance inflation factor (VIF) index. VIF value greater than 10 represented the presence of collinearity [62]; and 3) gait performance and demographic parameters were selected based on Akaike information criterion (AIC) values. Participants who exhibited no 20/30/40/50/60 s continuous walks were automatically categorized as frail in the respective model. All analyses were done using JMP (Version 11; SAS Institute Inc., Cary, NC, USA), and statistical significance was concluded when *p* < 0.05.

## Results

### Demographic and clinical measures

The study involved a total of 126 participants, among whom 44 were non-frail, 60 were pre-frail and 22 were frail according to the Fried frailty criteria [2]. Table 2 shows demographic and clinical characteristics. Pre-frail/frail participants were significantly older than non-frail participants, used assistive devices more frequently, had higher BMI, perceived tiredness, and fear of falling score (*p* < 0.05) and lower performance scores in ADL (*p* < 0.05).

**Table 2:** Demographic and clinical characteristics

**Characteristics**	**Non-frail** **(N)** **(*n*** **= 44)**			**Pre-frail/Frail (P/F)** **(*n*** **= 82)**			***p*-value**
							**N vs P/F**
Age (years)	74.6	±	6.5	81.2	±	8.6	**< 0.001**
Height (cm)	161.7	±	6.9	161.3	±	9.5	0.797
Weight (kg)	67.2	±	12.8	76.0	±	18.1	**0.005**
Body mass index	25.7	±	4.5	29.2	±	6.5	**0.002**
Gender							0.190
Male	6	(13.6)		19	(23.2)		
Female	38	(86.4)		63	(76.8)		
History of falls	13	(29.5)		37	(45.1)		0.085
Falls Efficacy Scale - International	20.8	±	4.2	31.3	±	11.6	**< 0.001**
Use of assistive devices	4	(9.1)		41	(50.0)		**< 0.001**
Mobility-tiredness scale	5.6	±	0.8	4.1	±	1.8	**< 0.001**
MMSE	29.2	±	1.1	28.6	±	1.6	0.060
CES-D	6.6	±	5.7	8.9	±	7.5	0.079
Barthel ADL Scale	97.6	±	4.6	93.8	±	7.9	**0.004**

Results presented as mean ± SD or number (%). Bold-faced values show statistical significance (*p* < 0.05)

### Sensor-based daily physical activity assessment

**Fig. 1:**
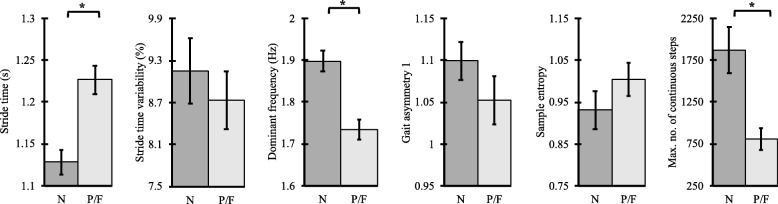
Comparison of continuous gait performance parameters between non-frail(N) and pre-frail(P)/frail(F) groups (*p < 0.05)

Table 3 shows the sensor-based DPA parameters for 60s continuous walking with resulting *p*-values of one-way ANOVA test (Fig. 1, Table 3) and Cohen’s d effect sizes. Supplementary Table 1 (a-d) shows the sensor-based DPA parameters for 20s, 30s, 40s, and 50s continuous walks with resulting *p*-values of one-way ANOVA tests respectively.

**Table 3:** Gait performance parameters for 60s continuous walks, one-way ANOVA results, and Cohen’s *d* effect sizes between non-frail(N) and pre-frail(P)/frail(F) groups

**Parameter**	**Non-frail** **(N)** **(*n*** **= 40)**			**Pre-frail/Frail** **(P/F)** **(*n*** **= 54)**			***p*-value (Eff. size)**
							**N vs. P/F**
*Temporal Gait Parameters*							
Step-time (s)	0.56	±	0.05	0.61	±	0.06	**< 0.001** (0.91)
Stride-time (s)	1.13	±	0.09	1.23	±	0.12	**< 0.001** (0.92)
*Time Domain Gait Variability*							
Step variability (%)	10.79	±	2.80	10.95	±	3.36	0.812 (0.05)
Stride variability (%)	9.16	±	2.94	8.74	±	3.04	0.509 (0.13)
*Frequency-domain Gait Variability*							
PSD max (W/Hz)	0.17	±	0.16	0.07	±	0.07	**< 0.001** (0.90)
PSD width (Hz)	0.22	±	0.10	0.21	±	0.03	0.446 (0.17)
PSD slope (W)	1.24	±	1.21	0.48	±	0.49	**< 0.001** (0.90)
Dominant frequency (Hz)	1.90	±	0.16	1.73	±	0.18	**< 0.001** (0.97)
*Gait Asymmetry*							
Asymmetry 1	1.10	±	0.14	1.05	±	0.21	0.229 (0.26)
Asymmetry 2	0.09	±	0.07	0.08	±	0.06	0.625 (0.10)
*Gait Irregularity*							
Time delay (ms)	145.39	±	14.90	156.25	±	23.86	0.124 (0.33)
Sample entropy (bits)	0.93	±	0.28	1.00	±	0.29	0.225 (0.25)
*Continuous Walk Quantitative Measures*							
Number of continuous walks	13.25	±	11.22	10.63	±	10.43	0.112 (0.33)
Total continuous walking duration (s)	4042.33	±	3012.86	2436.79	±	1988.46	**0.001** (0.70)
Max walking bout (s)	475.62	±	512.27	216.98	±	228.95	**0.001** (0.77)
Max number of continuous steps	1867.58	±	1735.98	896.63	±	1055.53	**0.001** (0.78)
Walking bout variability (%)	252.74	±	110.51	195.88	±	73.98	**0.002** (0.69)
Duration of non-continuous walks (% total of walking duration)	41.82	±	27.85	47.25	±	42.39	0.482 (0.15)

*PSD* Power Spectral Density

Of note, among the total 126 participants, 4 non-frail (9.09%), 17 pre-frail (28.33%), and 11 frail (50%) had no walking bout equal or longer than 60 s. These participants who did not have any 60s continuous walking bout (age: 81.59 ± 8.34) were significantly (*p* = 0.035) older than the participants who had continuous walks (age: 77.94 ± 8.43). Frailty status was also significantly (*p* = 0.001) different between the participants who did not have any continuous walking bouts (87.5% pre-frail/frail) compared to those who had continuous walks (57.45% pre-frail/frail). However, the BMI and gender were not significantly different between these groups (*p* > 0.079).

Among the 60s continuous gait parameters, frequency domain gait variability parameters and quantitative measures including maximum number of continuous steps, maximum walking bout and total continuous walking duration best discriminated non-frail and pre-frail/frail individuals. Step- and stride-time were significantly different between non-frail vs pre-frail/frail (*p* < 0.001). Further, among the frequency domain gait variability parameters, PSD amplitude, PSD slope, and the dominant frequency were significantly different between non-frail and pre-frail/frail groups (*p* < 0.05). However, step- and stride-time variabilities did not show statistical significance between the two groups (*p* > 0.1). Gait asymmetry, representing left and right step coordination, was not statistically significant between the two frailty groups (*p* > 0.1). Similarly, gait irregularity, measuring predictability of walking cycles, displayed an increasing trend towards pre-frailty/frailty; but the observed between-group differences were not significant (*p* > 0.1). All the continuous walk quantitative measures showed differences between the two groups, and most of these differences were statistically significant between the two groups (*p* < 0.05).

### Frailty prediction using gait performance parameters

**Fig. 2:**
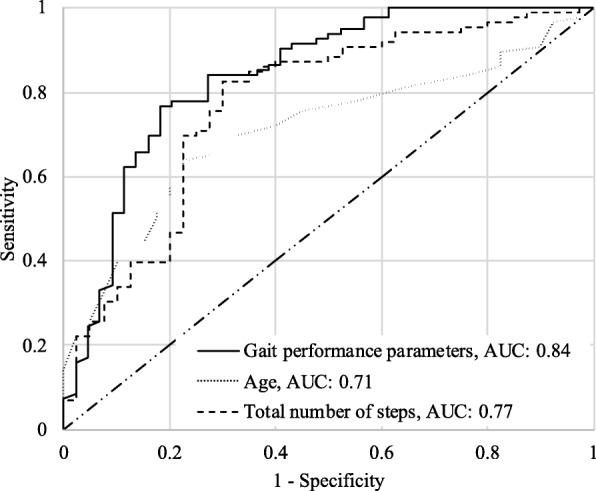
Logistic regression model ROC curves for age, total number of steps, and gait performance parameters

A multinomial logistic regression model was developed using gait performance parameters extracted from DPA along with age and BMI to predict frailty. A step-wise logistic model was developed with frailty groups as the dependent variable (non-frail vs. pre-frail/frail), for which, age (years), BMI (kg/m^2^), stride-time variability (%), dominant frequency (Hz), and maximum number of continuous steps were selected as independent variables (Table 4). The logistic regression model developed with these features was able to predict pre-frail/frail category with an improved receiver operating characteristics (ROC) area under curve (AUC) of 0.84 compared to age (ROC AUC: 0.71) and total number of steps for 48 h (ROC AUC: 0.77, Table 5). The ROC curves are shown in Fig. 2.

**Table 4:** Parameter estimates for logistic regression models developed with different parameters

**Model Features**	**Parameter**	**Parameter Estimate**	**Std. Error**	**χ2**	***p*-value**
MODEL 1: Age	Age	−0.0885	0.03	9.75	0.002
MODEL 2: Total number of steps	Total number of steps	0.0001	0.00	12.54	0.001
MODEL 3: Gait performance parameters	Age (years)	−0.1191	0.04	8.27	0.004
	BMI (kg/m^2^)	−0.1772	0.06	8.49	0.004
	Stride variability (%)	−0.2507	0.11	5.39	0.020
	Dominant frequency (Hz)	6.6265	2.14	9.62	0.002
	Max no. of continuous steps	0.0001	0.00	0.33	0.565

**Table 5:** Logistic model performance comparison of different parameters for 80% specificity

**Model Features**	**Accuracy**	**Sensitivity**	**Specificity**	**AUC**
**Age**	65.1%	58.1%	80%	0.71
**Total number of steps**	74.6%	46.5%	80%	0.77
**Gait performance parameters**	77.7%	76.8%	80%	0.84

Results of logistic regression models developed with gait performance parameters extracted from 20s, 30s, 40s and 50s continuous walks, age, and BMI to predict pre-frail/frail category for the different continuous walking lengths are shown in supplementary Table 2. The corresponding ROC curves in comparison with the 60s ROC curve are presented in supplementary Figure 1. Of note, we observed that among the five different durations (20s, 30s, 40s, 50s and 60s), gait performance parameters extracted from 60s continuous walks provided the best frailty assessment results (supplementary Table 2 and supplementary Figure 1).

## Discussion

As hypothesized, several sensor-based gait performance parameters significantly discriminated between non-frail and pre-frail/frail groups, even when adjusted with age. In our previous studies the total number of steps and walking duration could not significantly discriminate pre-frail participants from non-frail when adjusted with age [17]. This improvement in frailty assessment occurred due to utilization of the novel concept of continuous walking for obtaining both qualitative and quantitative gait performance parameters.

### Continuous walks

The ability to walk longer distances is instrumental for humans to perform various activities of daily living and lead an independent life. Walking requires complex mechanisms within the human sensory motor system to provide the necessary timing, coordination, and balance such that the interplay between the center of mass and the base of support are regulated in a repetitive manner [57, 63]. Previous studies that explored the gait characteristics of non-disabled adults for 2 weeks to define walking duration, found that 81% of all walking bouts lasted about 60 s [35, 36]. Also to assess the effect of continuous walk duration in pre-frailty/frailty assessment, we analyzed gait performance parameters extracted from 20s, 30s, 40s, 50s, and 60s continuous walking bouts and observed that 60s continuous walks provided the best pre-frailty/frailty assessment results compared to other continuous walk durations. Previous studies also showed that accelerometer-derived gait performance measures based on daily activities could improve fall risk evaluation in older adults, when 60-s continuous walking periods were implemented compared to overall number of steps [36]. Similarly, in the current study, 60 s continuous walking with no pauses resulted in better discrimination between the frailty groups (*p* < 0.05), when compared with continuous walks with shorter duration and walks that included pauses.

### Advantages of qualitative gait parameters

In addition to previously reported quantitative gait parameters (number of steps, mean walking bout duration, and longest walking bout duration) [17], here we extracted qualitative gait performance parameters (gait variability, asymmetry, and irregularity). Specifically, gait variability represented a promising measure for differentiating gait deficits among the three frailty categories. Gait variability, defined as the stride-to-stride fluctuation in walking cycles, has been previously associated with high risk of fall and cognitive impairments in elders [6, 45–47, 64]. Gait variability reflects inconsistency in physiological systems that regulate walking, including neuromuscular, reflexive postural control, and cardiovascular systems. We used two methods to assess gait variability: step/stride time variability using time-domain and power spectral density (PSD) using frequency-domain analyses. We observed that the frequency-domain parameters were significantly different between frailty groups, while time-domain parameters were not. Owing to the low sampling frequency in this study, some of the information content may be lost due to filtering for peak detection [65]. Hence, the PSD analysis performed on the entire raw acceleration signal may provide a more efficient tool for assessing gait variability for low sampling frequency motion sensor data.

Additionally, gait asymmetry and irregularity were also investigated here, as parameters that represent gait deficits independent of gait variability. Gait asymmetry, representing left-right step coordination, has been used as a metric to observe walking patterns in older individuals. Cognitive aging and sarcopenia render gait to be less automatic and left-right symmetry co-ordination is expected to require additional effort, especially in frail individuals [37]. Although gait asymmetry showed a decreasing trend from non-frail to pre-frail and frail samples, differences were not significant (Table 3). Since loss in step-coordination may also happen due to hip, knee, or ankle impairments [66], these confounding variables can mask the effect of frailty. Further, gait irregularity, representing the predictability of walking cycles, can be influenced by both neurological and neuromuscular diseases [18, 54–56]. Previous studies have used sample entropy to obtain gait predictability or repeatability to investigate differences in the relationship between executive function, and gait variability and stability during single and dual-task walking in persons with and without dementia [55]. We computed gait irregularity using the sample entropy method and observed an increase in irregularity in pre-frail/frail population but there was no statistical significance seen between the groups (Table 3).

### Limitations

There are limitations to consider in the interpretation of these findings. The first limitation is the lack of longitudinal validation of the DPA index for direct prediction of frailty-related health complications. Accordingly, due to a cross-sectional design of the current study, no conclusion can be made regarding the accuracy of the proposed index compared to the Fried test, or other types of frailty measures. Second limitation is adherence to the wearable sensor equipment. Though the participants did not express any obvious discomfort while wearing the sensors, it is possible that a few of the participants forgot to wear them immediately after a shower. Third, our sample was predominantly women. Although we did not observe a gender specific difference in gait performance, the model developed here may have limited generalizability to a population with a more balanced gender composition. Fourth, we consider a continuous walk as one with 20/30/40/50/60 s or longer duration with no pauses. However, it is possible that this walk was purposeful with the individual walking to a certain destination or that this walk was a random stroll with no specific purpose. These possibilities could potentially bias the quality of walking, but in our data we did not have the required information to categorize continuous walks as purposeful or random. Finally, not all participants, especially pre-frail and frail ones, had continuous walks during the 48 h of data collection, causing a reduced sample size for the pre-frail/frail group. To overcome this limitation, we combined pre-frail and frail participants and all the participants who did not exhibit continuous walking were automatically categorized as pre-frail/frail while developing the logistic model. Since detecting the onset of frailty at the pre-frail stage is most crucial in recovering health status [67], our findings suggested a promising method for pre-frailty identification using DPA data.

### Summary and conclusion

Using gait performance parameters extracted from 60s continuous walks within 48 h of daily monitoring, pre-frailty/frailty was identified with a sensitivity of 76.8% and specificity of 80% among elders. Findings suggest that these DPA-based 60s continuous walking parameters, including gait variability and the amount of continuous walking, may noticeably improve gait deficit assessment compared to previous in-clinic gait assessment methods. The proposed gait performance characterization based on sensor-based daily physical activity provides potential for being integrated into clinical care for in-home screening of frailty (much as a holter monitor is used) to provide information pertaining to an individual’s condition before hospital admission, or when frailty is suspected. This method is advantageous over its in-clinic counterpart as it is objective rather than subjective self-reported measures of physical activity, and is measured in real-world walking activities rather than an artificial clinical setting. Although Fried’s frailty criteria [2] provides an accurate frailty measure and we observed strong association with this gold standard, the DPA frailty index should be further validated within longitudinal settings for predicting adverse health outcomes among older adults. Nevertheless, our proposed technique eliminates the bias pertaining to self-reported measures, does not require the subject to commute to the clinic, and provides a continuous in-home assessment.

Healthcare research in wearable devices has been constantly growing in various areas like remote patient monitoring and healthcare [68–72], wearable sensor-based systems for health monitoring [73–76], and ambient-assisted living tools for older adults [77]. For the older adults requiring continuous health monitoring, sensor-based wearables and remote monitoring will help eliminate the hassle of periodic commute to diagnostic centers, reduce the amount of recurring admissions to the hospital, and facilitate more efficient clinical visits with objective results [78].

## Supplementary information

**Additional file 1: Supplementary Table 1**(a-d). Gait performance parameters for 20s, 30s, 40s and 50s continuous walks, one-way ANOVA results, and Cohen's d effect sizes between non-frail(N) and pre-frail(P)/frail(F) groups; **Supplementary Table 2.** Logistic model performance comparison using gait performance parameters between different duration of continuous walks for 80% specificity.

**Additional file 2: Supplementary Figure 1.** Logistic regression model receiver operating characteristic (ROC) curves for different continuous walk criteria (20, 30, 40, 50, and 60 second cutoff). Results are presented for predictions using gait performance models (gait performance parameters, age, and BMI).

## Data Availability

The datasets used and/or analyzed during the current study available from the corresponding author on reasonable request.

## Abbreviations

ADL: Barthel activity of daily living
AIC: Akaike information criteria
ANOVA: Analysis of variance
AUC: Area under the curve
BMI: Body mass index
CES-D: Center for Epidemiologic Studies Depression Scale
CGA: Comprehensive Geriatric Assessment
DPA: Daily physical activity
MMSE: Mini-mental state examination
PSD: Power spectral density
ROC: Receiver operating characteristic
SampEn: Sample entropy
SD: Standard deviation
VIF: Variance inflation factor
WHO: World health organization
