# Supplementary material for: Sensor-based characterization of daily walking: a new paradigm in pre-frailty/frailty assessment
Source: BMC Geriatr. 2020 May 6;20:164. doi: 10.1186/s12877-020-01572-1 (PMC7203790; doi:10.1186/s12877-020-01572-1)
Supplement: Supplementary file 1 — Additional file 1: Supplementary Table 1(a-d). Gait performance parameters for 20s, 30s, 40s and 50s continuous walks, one-way ANOVA results, and Cohen's d effect sizes between non-frail(N) and pre-frail(P)/frail(F) groups; Supplementary Table 2. Logistic model performance comparison using gait performance parameters between different duration of continuous walks for 80% specificity. [file 12877_2020_1572_MOESM1_ESM.docx]

**Supplementary Table 1(a).** Gait performance parameters for 20s continuous walks, one-way ANOVA results, and Cohen’s *d* effect sizes between non-frail(N) and pre-frail(P)/frail(F) groups

| **Parameter** | **Non-frail (N)**  **(n=42)** | | | **Pre-frail/Frail**  **(P/F)**  **(n=74)** | | | ***p*-value (Effect size)** |
| --- | --- | --- | --- | --- | --- | --- | --- |
|  |  |  |  |  |  |  | **N vs P/F** |
| Temporal Gait Parameters  Step-time (s)  Stride-time (s) |  |  |  |  |  |  |  |
|  | 0.58 | ± | 0.03 | 0.62 | ± | 0.05 | **<0.001**(1.00) |
|  | 1.16 | ± | 0.07 | 1.25 | ± | 0.10 | **<0.001**(1.03) |
| Time Domain Gait Variability  Step variability (%)  Stride variability (%) |  |  |  |  |  |  |  |
|  | 13.09 | ± | 1.79 | 12.33 | ± | 2.56 | 0.090(0.35) |
|  | 11.20 | ± | 1.85 | 10.00 | ± | 2.37 | **0.006**(0.56) |
| Frequency-domain Gait Variability  PSD max (W/Hz)  PSD width (Hz)  PSD slope (W)  Dominant frequency (Hz) |  |  |  |  |  |  |  |
|  | 0.11 | ± | 0.07 | 0.05 | ± | 0.05 | **<0.001**(0.97) |
|  | 0.22 | ± | 0.03 | 0.22 | ± | 0.03 | 0.9212(0.02) |
|  | 0.75 | ± | 0.56 | 0.34 | ± | 0.34 | **<0.001**(0.97) |
|  | 1.87 | ± | 0.12 | 1.75 | ± | 0.18 | **<0.001**(0.78) |
| Gait Asymmetry  Asymmetry 1  Asymmetry 2 |  |  |  |  |  |  |  |
|  | 1.13 | ± | 0.13 | 1.02 | ± | 0.21 | **0.003**(0.63) |
|  | 0.11 | ± | 0.05 | 0.13 | ± | 0.16 | 0.433(0.19) |
| Gait Irregularity  Time delay (ms)  Sample entropy (bits) |  |  |  |  |  |  |  |
|  | 144.74 | ± | 11.04 | 150.38 | ± | 18.57 | 0.076(0.38) |
|  | 0.71 | ± | 0.18 | 0.83 | ± | 0.26 | **0.008**(0.55) |
| Continuous Walk Quantitative Measures  Number of continuous walks  Total continuous walking duration (s)  Max walking bout (s)  Max number of continuous steps  Walking bout variability (%) |  |  |  |  |  |  |  |
|  | 45.29 | ± | 29.54 | 40.69 | ± | 33.48 | 0.460(0.15) |
|  | 3208.15 | ± | 2715.09 | 1865.63 | ± | 1760.84 | **0.002**(0.60) |
|  | 441.28 | ± | 502.71 | 166.52 | ± | 189.18 | **<0.001**(0.79) |
|  | 1771.21 | ± | 1716.76 | 710.59 | ± | 866.15 | **<0.001**(0.82) |
|  | 99.01 | ± | 68.60 | 60.58 | ± | 37.32 | **<0.001**(0.73) |
| Duration of non-continuous walks (% of total walking duration) | 72.83 | ± | 17.53 | 74.49 | ± | 23.17 | 0.688(0.08) |

**Supplementary Table 1(b).** Gait performance parameters for 30s continuous walks, one-way ANOVA results, and Cohen’s *d* effect sizes between non-frail(N) and pre-frail(P)/frail(F) groups

| **Parameter** | **Non-frail (N)**  **(n=41)** | | | **Pre-frail/Frail**  **(P/F)**  **(n=70)** | | | ***p*-value (Effect size)** |
| --- | --- | --- | --- | --- | --- | --- | --- |
|  |  |  |  |  |  |  | **N vs P/F** |
| Temporal Gait Parameters  Step-time (s)  Stride-time (s) |  |  |  |  |  |  |  |
|  | 0.58 | ± | 0.03 | 0.61 | ± | 0.05 | **<0.001**(0.83) |
|  | 1.16 | ± | 0.05 | 1.22 | ± | 0.09 | **<0.001**(0.85) |
| Time Domain Gait Variability  Step variability (%)  Stride variability (%) |  |  |  |  |  |  |  |
|  | 12.27 | ± | 1.53 | 11.81 | ± | 2.51 | 0.290(0.23) |
|  | 10.59 | ± | 1.65 | 9.64 | ± | 2.43 | **0.023**(0.47) |
| Frequency-domain Gait Variability  PSD max (W/Hz)  PSD width (Hz)  PSD slope (W)  Dominant frequency (Hz) |  |  |  |  |  |  |  |
|  | 0.12 | ± | 0.08 | 0.06 | ± | 0.05 | **<0.001**(0.91) |
|  | 0.22 | ± | 0.02 | 0.22 | ± | 0.02 | 0.357(0.10) |
|  | 0.85 | ± | 0.60 | 0.42 | ± | 0.33 | **<0.001**(0.92) |
|  | 1.87 | ± | 0.10 | 1.78 | ± | 0.17 | **<0.001**(0.73) |
| Gait Asymmetry  Asymmetry 1  Asymmetry 2 |  |  |  |  |  |  |  |
|  | 1.10 | ± | 0.08 | 1.04 | ± | 0.13 | **0.013**(0.54) |
|  | 0.10 | ± | 0.04 | 0.11 | ± | 0.06 | 0.334(0.21) |
| Gait Irregularity  Time delay (ms)  Sample entropy (bits) |  |  |  |  |  |  |  |
|  | 143.39 | ± | 15.12 | 150.81 | ± | 18.26 | **0.030**(0.44) |
|  | 0.70 | ± | 0.15 | 0.79 | ± | 0.18 | **0.012**(0.52) |
| Continuous Walk Quantitative Measures  Number of continuous walks  Total continuous walking duration (s)  Max walking bout (s)  Max number of continuous steps  Walking bout variability (%) |  |  |  |  |  |  |  |
|  | 52.15 | ± | 31.61 | 43.16 | ± | 35.05 | 0.237(0.27) |
|  | 4792.57 | ± | 3713.61 | 2931.11 | ± | 2754.55 | **0.003**(0.58) |
|  | 581.30 | ± | 536.88 | 287.67 | ± | 275.13 | **<0.001**(0.72) |
|  | 2454.10 | ± | 1970.72 | 1249.83 | ± | 1414.70 | <**0.001**(0.71) |
|  | 102.49 | ± | 66.00 | 67.07 | ± | 41.52 | **<0.001**(0.66) |
| Duration of non-continuous walks (% of total walking duration) | 57.01 | ± | 45.39 | 60.82 | ± | 43.54 | 0.662(0.09) |

**Supplementary Table 1(c).** Gait performance parameters for 40s continuous walks, one-way ANOVA results, and Cohen’s *d* effect sizes between non-frail(N) and pre-frail(P)/frail(F) groups

| **Parameter** | **Non-frail (N)**  **(n=41)** | | | **Pre-frail/Frail**  **(P/F)**  **(n=68)** | | | ***p*-value (Effect size)** |
| --- | --- | --- | --- | --- | --- | --- | --- |
|  |  |  |  |  |  |  | **N vs P/F** |
| Temporal Gait Parameters  Step-time (s)  Stride-time (s) |  |  |  |  |  |  |  |
|  | 0.57 | ± | 0.03 | 0.60 | ± | 0.05 | **0.001**(0.69) |
|  | 1.15 | ± | 0.06 | 1.20 | ± | 0.09 | **0.001**(0.71) |
| Time Domain Gait Variability  Step variability (%)  Stride variability (%) |  |  |  |  |  |  |  |
|  | 11.41 | ± | 1.26 | 11.63 | ± | 2.13 | 0.557(0.13) |
|  | 9.74 | ± | 1.12 | 9.64 | ± | 2.03 | 0.754(0.07) |
| Frequency-domain Gait Variability  PSD max (W/Hz)  PSD width (Hz)  PSD slope (W)  Dominant frequency (Hz) |  |  |  |  |  |  |  |
|  | 0.13 | ± | 0.10 | 0.08 | ± | 0.06 | **0.001**(0.63) |
|  | 0.22 | ± | 0.04 | 0.21 | ± | 0.02 | 0.162(0.27) |
|  | 0.95 | ± | 0.76 | 0.58 | ± | 0.44 | **0.002**(0.62) |
|  | 1.87 | ± | 0.12 | 1.79 | ± | 0.15 | **0.002**(0.64) |
| Gait Asymmetry  Asymmetry 1  Asymmetry 2 |  |  |  |  |  |  |  |
|  | 1.09 | ± | 0.06 | 1.04 | ± | 0.07 | <**0.001**(0.75) |
|  | 0.09 | ± | 0.03 | 0.09 | ± | 0.04 | 0.879(0.03) |
| Gait Irregularity  Time delay (ms)  Sample entropy (bits) |  |  |  |  |  |  |  |
|  | 141.72 | ± | 16.25 | 148.64 | ± | 19.81 | 0.062(0.38) |
|  | 0.72 | ± | 0.17 | 0.82 | ± | 0.19 | **0.006**(0.56) |
| Continuous Walk Quantitative Measures  Number of continuous walks  Total continuous walking duration (s)  Max walking bout (s)  Max number of continuous steps  Walking bout variability (%) |  |  |  |  |  |  |  |
|  | 44.27 | ± | 31.86 | 39.87 | ± | 27.44 | 0.447(0.15) |
|  | 4786.99 | ± | 3396.08 | 3638.56 | ± | 2767.16 | 0.05(0.35) |
|  | 674.54 | ± | 529.17 | 490.87 | ± | 396.81 | **0.042**(0.40) |
|  | 2585.07 | ± | 1882.39 | 1701.93 | ± | 1431.70 | **0.007**(0.53) |
|  | 118.95 | ± | 62.98 | 96.64 | ± | 52.49 | **0.049**(0.39) |
| Duration of non-continuous walks (% of total walking duration) | 51.85 | ± | 26.85 | 53.45 | ± | 31.51 | 0.0.787(0.05) |

**Supplementary Table 1(d).** Gait performance parameters for 50s continuous walks, one-way ANOVA results, and Cohen’s *d* effect sizes between non-frail(N) and pre-frail(P)/frail(F) groups

| **Parameter** | **Non-frail (N)**  **(n=41)** | | | **Pre-frail/Frail**  **(P/F)**  **(n=67)** | | | ***p*-value (Effect size)** |
| --- | --- | --- | --- | --- | --- | --- | --- |
|  |  |  |  |  |  |  | **N vs P/F** |
| Temporal Gait Parameters  Step-time (s)  Stride-time (s) |  |  |  |  |  |  |  |
|  | 0.57 | ± | 0.03 | 0.59 | ± | 0.04 | **0.005**(0.59) |
|  | 1.14 | ± | 0.06 | 1.18 | ± | 0.08 | **0.004**(0.60) |
| Time Domain Gait Variability  Step variability (%)  Stride variability (%) |  |  |  |  |  |  |  |
|  | 11.30 | ± | 1.47 | 11.67 | ± | 2.32 | 0.365(0.19) |
|  | 9.60 | ± | 1.42 | 9.72 | ± | 2.20 | 0.763(0.06) |
| Frequency-domain Gait Variability  PSD max (W/Hz)  PSD width (Hz)  PSD slope (W)  Dominant frequency (Hz) |  |  |  |  |  |  |  |
|  | 0.14 | ± | 0.10 | 0.09 | ± | 0.06 | **0.001**(0.65) |
|  | 0.23 | ± | 0.05 | 0.21 | ± | 0.02 | **0.009**(0.52) |
|  | 0.99 | ± | 0.80 | 0.61 | ± | 0.40 | **0.001**(0.63) |
|  | 1.87 | ± | 0.12 | 1.79 | ± | 0.14 | **0.002**(0.64) |
| Gait Asymmetry  Asymmetry 1  Asymmetry 2 |  |  |  |  |  |  |  |
|  | 1.09 | ± | 0.08 | 1.04 | ± | 0.06 | <**0.001**(0.78) |
|  | 0.09 | ± | 0.05 | 0.08 | ± | 0.03 | 0.223(0.24) |
| Gait Irregularity  Time delay (ms)  Sample entropy (bits) |  |  |  |  |  |  |  |
|  | 145.41 | ± | 16.14 | 153.70 | ± | 17.09 | **0.014**(0.50) |
|  | 0.73 | ± | 0.17 | 0.81 | ± | 0.20 | **0.031**(0.44) |
| Continuous Walk Quantitative Measures  Number of continuous walks  Total continuous walking duration (s)  Max walking bout (s)  Max number of continuous steps  Walking bout variability (%) |  |  |  |  |  |  |  |
|  | 35.00 | ± | 22.56 | 35.58 | ± | 19.82 | 0.889(0.03) |
|  | 4938.44 | ± | 3808.23 | 4314.07 | ± | 2612.10 | 0.315(0.19) |
|  | 565.05 | ± | 517.94 | 500.43 | ± | 368.54 | 0.451(0.15) |
|  | 2303.76 | ± | 1791.12 | 1758.58 | ± | 1353.01 | 0.076(0.35) |
|  | 97.60 | ± | 53.18 | 93.54 | ± | 45.33 | 0.674(0.08) |
| Duration of non-continuous walks (% of total walking duration) | 45.38 | ± | 28.84 | 44.72 | ± | 62.72 | 0.155(0.01) |

**Supplementary Table 2.** Logistic model performance comparison using gait performance parameters between different duration of continuous walks for 80% specificity

| **Continuous walk duration** | **N (n)** | **P/F (n)** | **Accuracy** | **Sensitivity** | **Specificity** | **AUC** |
| --- | --- | --- | --- | --- | --- | --- |
| **20s** | 42 | 74 | 74.6% | 62.0% | 80% | 0.78 |
| **30s** | 41 | 70 | 76.1% | 65.8% | 80% | 0.79 |
| **40s** | 41 | 68 | 76.9% | 60.8% | 80% | 0.76 |
| **50s** | 41 | 67 | 75.4% | 51.25 | 80% | 0.75 |
| **60s** | 40 | 54 | 77.7% | 76.8% | 80% | 0.84 |

**Figure Title:**

**Supplementary Figure 1.** Logistic regression model receiver operating characteristic (ROC) curves for different continuous walk criteria (20, 30, 40, 50, and 60 second cutoff). Results are presented for predictions using gait performance models (gait performance parameters, age, and BMI).
