# Supplementary figures and images for: Sensor-based characterization of daily walking: a new paradigm in pre-frailty/frailty assessment
Source: BMC Geriatr. 2020 May 6;20:164. doi: 10.1186/s12877-020-01572-1 (PMC7203790; doi:10.1186/s12877-020-01572-1)

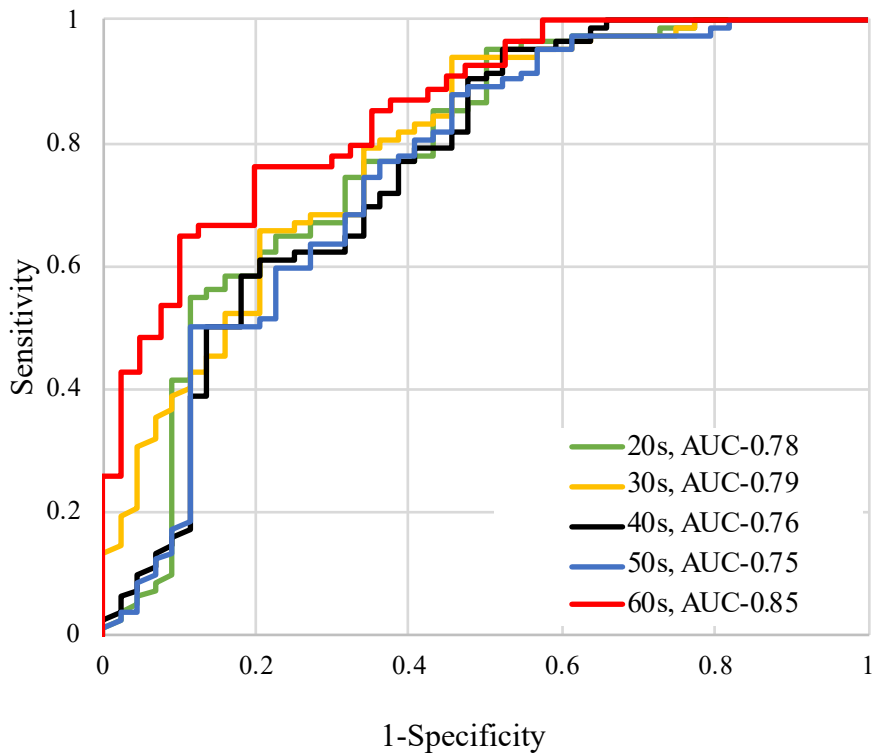

Supplement: Supplementary file 2 — Additional file 2: Supplementary Figure 1. Logistic regression model receiver operating characteristic (ROC) curves for different continuous walk criteria (20, 30, 40, 50, and 60 second cutoff). Results are presented for predictions using gait performance models (gait performance parameters, age, and BMI). [file 12877_2020_1572_MOESM2_ESM.pdf]
